# Thermoregulatory and Metabolic Demands of Naval Special Warfare Divers During a 6-h Cold-Water Training Dive

**DOI:** 10.3389/fphys.2021.674323

**Published:** 2021-09-29

**Authors:** Andrea C. Chapin, Laura J. Arrington, Jake R. Bernards, Karen R. Kelly

**Affiliations:** ^1^Applied Translational Exercise and Metabolic Physiology Team, Warfighter Performance, Naval Health Research Center, San Diego, CA, United States; ^2^Leidos, Inc., San Diego, CA, United States

**Keywords:** energy expenditure, metabolism, thermoregulation, submersion, dive response, substrate utilization

## Abstract

**Introduction:** Extreme environmental conditions induce changes in metabolic rate and substrate use due to thermoregulation. Cold-water full-body submersion for extended periods of time is inevitable for training and missions carried out by Naval Special Warfare divers. Anthropometric, physiologic, and metabolic data have been reported from partial immersion in cold water in non-thermally protected men; data is limited in thermally protected divers in extremely cold water. Thermoregulatory and metabolic demands during prolonged cold-water submersion in Naval Special Warfare divers are unknown.

**Objective:** Assess thermoregulatory and metabolic demands of Naval Special Warfare divers surrounding prolonged cold-water submersion.

**Materials and Methods:** Sixteen active-duty U.S. Navy Sea Air and Land (SEAL) operators tasked with cold-water dive training participated. Divers donned standard military special operations diving equipment and fully submerged to a depth of ∼ 6 m in a pool chilled to 5°C for a 6-h live training exercise. Metabolic measurements were obtained via indirect calorimetry for 10-min pre-dive and 5-min post dive. Heart rate, skin temperature, and core temperature were measured throughout the dive.

**Results:** Core temperature was maintained at the end of the 6-h dive, 36.8 ± 0.4°C and was not correlated to body composition (body fat percentage, lean body mass) or metabolic rate. SEALs were not at risk for non-freezing cold injuries as mean skin temperature was 28.5 ± 1.6°C at end of the 6-h dive. Metabolic rate (kcal/min) was different pre- to post-dive, increasing from 1.9 ± 0.2 kcal/min to 2.8 ± 0.2 kcal/min, *p* < 0.001, 95% CI [0.8, 1.3], Cohen’s d effect size 2.3. Post-dive substrate utilization was 57.5% carbohydrate, 0.40 ± 0.16 g/min, and 42.5% fat, 0.13 ± 0.04 g/min.

**Conclusion:** Wetsuits supported effective thermoprotection in conjunction with increase in thermogenesis during a 6-h full submersion dive in 5°C. Core temperature was preserved with an expected decrease in skin temperature. Sustained cold-water diving resulted in a 53% increase in energy expenditure. While all participants increased thermogenesis, there was high inter-individual variability in metabolic rate and substrate utilization. Variability in metabolic demands may be attributable to individual physiologic adjustments due to prior cold exposure patterns of divers. This suggests that variations in metabolic adjustments and habituation to the cold were likely. More work is needed to fully understand inter-individual metabolic variability to prolonged cold-water submersion.

## Introduction

Military divers Perform training and operations in harsh environments that impose profound physiological strain to human systems, including extreme cold and aquatic elements. Mission success and ultimately survival, depend on maintenance of thermal balance—which is a function of the preservation of heat loss and thermogenesis (Stocks et al., 2004). Behavioral modifications, such as wearing wetsuits, help mitigate the stress imposed from the exposure to cold-water by supporting thermoregulation and preventing hypothermia (Shiraki et al., 1986; Cotter and Taylor, 1995; Pendergast and Mollendorf, 2011). However, wetsuits alone only reduce heat dissipation from the body without eliminating physiological risk (Hayward and Eckerson, 1984; Arieli et al., 1997; Wakabayashi et al., 2006; Naebe et al., 2013). Individuals remain susceptible to hypothermia—especially as the duration of cold exposure persists.

In conjunction to the prevention of heat loss through insulative properties (e.g., fat mass, muscle) and wetsuits, thermal balance is also supported by generation of heat through increased physical activity, shivering, and non-shivering thermogenesis (Castellani and Young, 2016; Haman and Blondin, 2017). Heat production increases metabolic demand independent of the modality, although cold exposure can play an important role. Metabolic rate has been documented to increase up to fivefold over resting rate baselines in cold exposure (Sramek et al., 2000; Haman, 2006; Brychta and Chen, 2017). This demonstrates the importance of interactions such as physical activity and environmental temperature; however, several questions remain unanswered if addressed to a military population. Military operations often require both long sedentary periods interspersed with short bursts of intense physical activity. Further, military cold exposure is often by design, due to mission requirements, rather than by accidental exposure. Thus, there is a need to understand thermoregulation in this context, in order to reduce risk of hypothermia, prepare for cold water trainings and operations and maintain ability to perform.

Moreover, the point about controlled versus uncontrolled exposure raises questions about specific applications with military relevance. Most previous examination of cold-water immersion and cold-exposure have been conducted in controlled laboratory environments. The goal has been to understand survivability during times of accidental exposure. These efforts were aimed at determining time to hypothermia in accidents such as falling off a vessel at sea or through broken ice on a lake (Hayward and Eckerson, 1984; Tipton et al., 1991; Jacobs, 1996). These controlled circumstances do not readily lend themselves to conclusions for similar encounters occurring during military operations. For instance, laboratory tasks often involve nude or semi-nude diving whereas military divers wear wetsuits or drysuits to protect themselves from the environments while conducting work (Hayward and Eckerson, 1984). Furthermore, these studies are conducted over relatively short durations of time (60–180 min) in average cold water temperatures (10–12°C) to simulate time before a rescue, often sitting in a tub or pool where there is no environmental effect such as current or wind (Martineau and Jacobs, 1988, 1989; Glickman-Weiss et al., 1994; Castellani et al., 1998; Sramek et al., 2000). Furthermore, many of these studies are also conducted as water-immersion studies, where the head of the participant remains out of water, compared to submersion studies where the entire participant is submerged (Sramek et al., 2000; Stocks et al., 2004; Xu and Giesbrecht, 2018). Any one methodological influence could complicate extrapolation and inference derived from these studies when considering their results for military divers.

Another variable to consider is individual differences in thermoregulation. The majority of variance between individuals has been attributed to body composition (e.g., body fat percentage) and body size (Castellani and Young, 2016). While all tissue provides insulation, adipose provides the highest thermal protection (Carlson et al., 1958). High subcutaneous fat levels defend against heat loss through the reduction in conductive heat transfer from deeper tissues during cold-water immersion (Hayward and Keatinge, 1981; Bourdon et al., 1995). Therefore, larger stature individuals might be at a greater risk of hypothermia with prolonged exposure due to their higher surface-to-mass ratio coupled to the conductive properties of water which readily moves body heat away (Gagge and Gonzalez, 2011). One understudied principle involves whether similar thermoregulatory responses are observed when larger or particularly fit individuals, such as military personal, don protective gear.

Thus, the current study was designed to address the gap in understanding related to thermoregulation and metabolic demand while submerged in thermoprotective gear in extreme cold-water (<10°C). We measured metabolic rate, substrate partitioning and effect on thermoregulation in Naval Special Warfare (NSW) divers during an operationally relevant (mission task oriented) 6-h practical training dive in 5°C water. We hypothesized that individuals with more body fat would require less energy to maintain thermo-homeostasis and that divers would rely more heavily on fat due to thermoprotecive gear and decreased reliance on shivering to maintain body temperature.

## Materials and Methods

### Ethics Statement

This study was approved by the Institutional Review Board at Naval Health Research Center and adhered to Department of the Navy human research protection policies (Protocol NHRC.2017.0019). All participants provided written informed consent.

### Participants

A total of sixteen male active-duty U.S. Navy Sea Air and Land (SEAL) operators that were tasked with cold-water training participated in this study.

### Diving Equipment

Divers donned standard military special operations diving equipment for cold water conditions. Wetsuits (Yazbeck, Redondo Beach, CA) were new (never worn), made of closed cell 10-mm thick neoprene. Thermal insulation properties have not been evaluated; however, at depths less than 30 feet, there is no compression of the material due to their closed-cell design. The “Farmer John” configuration consisted of a two-piece garment; the underlayer was long neoprene pants (to the ankles) with a sleeveless neoprene top. Over this, the second layer was a long-sleeve neoprene top (to the wrists) with a beaver tail, resulting in 20-mm of neoprene covering the torso. Divers wore two layers of 5-mm thick gloves and booties, for 10 mm of protection on hands and feet. Breathing gas was supplied to divers using closed-circuit oxygen rebreathers to eliminate bubbles (Draeger Inc., Houston, Texas). The LAR Draeger apparatus provides 100% oxygen to the diver, recycling expelled breath into a closed circuit where it is filtered for carbon-dioxide.

### Diving Procedure

Data was collected over the course of 4 days in a chilled training pool on a military base. Each day, participants arrived at the pool at 0700 in the morning after an overnight fast. Divers were instructed to follow their typical diet and not consume any alcohol prior to the overnight fast. After pre-dive measurements, divers were permitted to eat prior to the 6-h dive. Due to being submerged, divers did not ingest any food during the 6-h dive period. None of the divers participated in strenuous exercise the morning of the dive; however, one used a bicycle for transportation to the pool. Sleep information was not obtained. Following equipment checks, divers submersed at 0900 to a depth of 6 m (0.6 ATM) in a pool chilled to 5°C for 6-h. Air in the testing room was 31°C. Divers sat passive at the bottom of the pool except for occasionally surfacing for the training, keeping rebreathers on. At dive cessation, immediately upon surfacing and before exiting the cold pool, Draeger rebreathers were removed and replaced with a Hans Rudolph molded silicone rubber mouthpiece connected to a two-way non-rebreathing valve (Hans Rudolph Inc., Shawnee, KS, United States), and nose clip, for 5-min respiratory gas collection.

### Metabolic Measurements

Prior to donning dive gear and submerging into the pool, divers sat quietly for expired respiratory gas collection for metabolic measurements via indirect calorimetry for 10 min (Parvo Medics, Salt Lake City, Utah). Post-dive, gas collection via indirect calorimetry was performed immediately upon divers surfacing. Divers came up in pairs and remained in the pool for gas collection. At the surface, one remained submerged and the other sat on the swim step, removed the regulator mouthpiece and inserted the Rudolph mouthpiece for 5-min. Metabolic data from indirect calorimetry pre-dive and post-dive were summarized into a digital data file and exported to Microsoft Excel. Average values were determined from each pre-dive and post-dive measurement periods for absolute VO_2_ (L/min), relative VO_2_ (ml/kg/min), Respiratory Exchange Ratio (RER), carbohydrate utilization (kcal/min), fat utilization (kcal/min). Calories per minute from carbohydrate and fat were divided by 4 and 9, respectively to determine grams per minute of each substrate. Metabolic rate (kcal/min) was calculated by multiplying VO_2_ (L/min) by the thermal equivalent of oxygen (Zuntz, 1901). Validity of RER was assessed by examining F_*E*_CO_2_ %, and the ventilatory equivalent for carbon dioxide (VE/VCO_2_), to ensure RER reflected non-protein substrate utilization and not ventilatory changes (Mezzani, 2017). Resting metabolic rate (RMR) was determined using the Cunningham equation (Schofield et al., 2019) for the 12 participants that had body composition measured. Harris-Benedict equation was used to estimate RMR when fat free mass data was not available. VO_2_ max was obtained in a laboratory using indirect calorimetry (Parvo Medics, Salt Lake City, Utah). Participants performed a modified Balke test on a treadmill as previously described (Jensen et al., 2016). Maximal effort parameters were a plateau in oxygen consumption despite increasing workload a RER greater than 1.05–1.15 and lactate at or above 8 mM.

### Anthropometric Measurements

Body composition was obtained using Dual-Energy X-ray Absorptiometry (DXA) total body tissue quantitation (Lunar Prodigy, GE Healthcare, Madison, WI). Weight was measured using a calibrated digital scale to the nearest 0.01 kg (SECA, Germany). Height was measured to the nearest 0.01 cm using a stadiometer (SECA, Germany).

### Physiological Monitoring

Heart rate was continuously measured throughout the dive using Polar Team Pro Sensors (Polar Electro, Bethpage, New York). Prior to the dive, individual profiles were created with participant information. Polar software provided estimated calorie expenditure data from predictive equations using collected data and participant demographic information.

### Thermoregulation

The night before data collection, single use ingestible core temperature capsules (Vital Sense, Philips Respironics, Bend, Oregon) were activated and distributed with instructions to ingest the capsule at 0500 on the day of the dive, which was approximately 4 h prior to dive time. Thermistor-based capsules have a sensing range of 25–50°C with reported accuracy ± 0.1°C. Capsules pass through the GI tract without affecting bodily functions and are easily passed. Immediately prior to dive, skin temperature patches (Vital Sense, Philips Respironics, Bend, Oregon) were all affixed to the right side of the participant at the following sites: dorsal hand, dorsal foot, mid pectoralis (chest), lateral deltoid (arm), mid-thigh (thigh), and mid-calf (leg). Reported accuracy of the skin patches was ± 0.25°C (−20 to 32°C). Mean skin temperature was calculated using four of the six sites; chest, arm, thigh, and leg (Ramanathan, 1964). Skin temperature was measured every 8 min and core temperature was measured every minute throughout the dive. Temperature values are reported from each hour of the dive. Termination criteria was 10°C skin temperature or 35°C core temperature.

#### Equations

Mean skin temperature (Ramanathan, 1964)


$${\text{T}}_{sk}=0.3\left({\text{T}}_{\text{chest}}+T_{\mathrm{arm}}\right)+0.2\left({\text{T}}_{\mathrm{thigh}}+{\text{T}}_{\mathrm{leg}}\right)$$

Cold Strain Index (Moran et al., 1999)


$$\text{CSI}=6.67\left({\text{T}}_{\mathrm{coret}}-{\text{T}}_{\mathrm{core0}}\right)\times {\left(35-{\text{T}}_{\mathrm{core0}}\right)}^{-1}+3.33\left({\text{T}}_{\mathrm{skt}}-{\text{T}}_{\mathrm{sk0}}\right){\left(20-{\text{T}}_{\mathrm{sk0}}\right)}^{-1}$$

Where T_*core*0_ and T_*sk*0_ are the initial measurements and T_*coret*_ and T_*skt*_ are simultaneous measurements taken at any time *t*; when T_coret_ > T_core0_, then T_coret_–T_core 0_ = 0.

Cold Strain Index (CSI) was first described by Moran et al. (1999). CSI was developed as an analogous tool to the physiological strain index, but to be used in response to cold environments and capable of indicating cold strain in real time (Moran et al., 1999). CSI is a weighted average of core temperature and mean skin temperature differences from initial baseline values, rated 1–10 where 1–2 is little to no cold strain, 3–4 is low cold strain, 5–6 is moderate cold strain, 7–8 is high cold strain, and 9–10 is very high cold strain.

Body Surface Area (BSA; m^2^) (DuBois and DuBois, 1916)


$$\text{BSA}=0.007184x(\text{height}{\left(\text{m}\right)}^{0.725}\times \text{weigh}t{\left(\mathrm{kg}\right)}^{0.425}$$

Harris-Benedict equation (Jagim et al., 2018)


RMR=66.47+(13.75×W)∔(5×H)-(6.76×A)

Equation to predict resting metabolic rate where W = weight in kilograms, H = height in centimeters, and A = age in years.

Cunningham equation (Cunningham, 1980)


RMR=500+22(LBM)

Equation to predict resting metabolic rate where LBM = lean body mass in kilograms.

### Statistical Analysis

Statistical analysis was conducted using IBM Statistical Package for Social Science (SPSS) Version 26 (IBM, Armonk, New York, United States). Central tendency and dispersion are reported as the mean ± standard deviation. All pre- to post-dive variables were analyzed using paired samples *t*-tests. Confidence intervals are reported at the 95% level for change of the mean difference pre-dive to post-dive. Effect size was calculated for *t*-tests by dividing the mean difference by the standard deviation of the difference (Cohen’s d) (Cohen, 1988, 1992). Pearson correlation was used to assess relationships between body composition and metabolic variables. Effect size was calculated for correlations using the coefficient of determination, r^2^. Significance for all analyses were accepted at *P* < 0.05. One diver (participant 11) was excluded from metabolic data analysis due to hyperventilation during gas collection post-dive which presented as an abnormally high VE/VCO_2_.

## Results

Participant demographic and anthropometric data are to be found in Table 1.

**TABLE 1:** Participant demographics.

	**n**	**Mean ± SD**	**Range**
Age (years)	16	29.6 ± 2.9	24–33
Height (cm)	16	178.8 ± 4.1	167.6–185.4
Weight (kg)	16	88.8 ± 7.1	77.3–97.7
Body mass index (kg/m^2^)	16	27.7 ± 1.7	25.0–30.1
Body surface area (m^2^)	16	2.07 ± 0.1	1.87–2.20
Body fat %	12	18.5 ± 3.3	13.8–24
Lean mass %	11	77.0 ± 0.03	73–84
Fat free mass (kg)	12	73.4 ± 6.9	63.0–82.9
Lean tissue mass (kg)	11	69.5 ± 6.2	57.1–78.4
VO_2_ max (ml/kg/min)	9	55.2 ± 6.6	43.9–63.1
RMR* cunningham (kcals/day)	12	2,115 ± 151	1,886–2,324
RMR* HARRIS-benedict (kcals/day)	16	1,981 ± 109	1,744–2,108

*RMR, resting metabolic rate.

*Where n < 16, participants missed body composition or VO_2_ max testing due to schedule change and duty obligations.*

### Temperature

**FIGURE 1:**
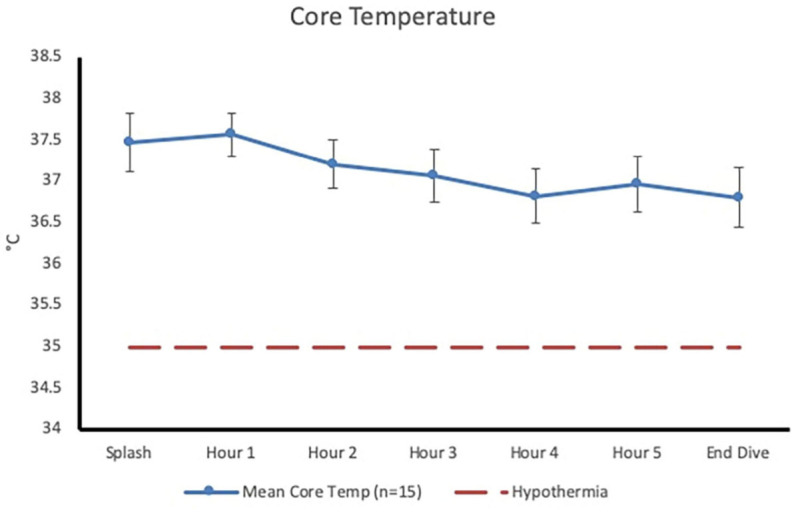
Core temperature (mean ± SD) at dive splash and each subsequent hour of the dive. Dashed line represents clinical hypothermia.

**FIGURE 2:**
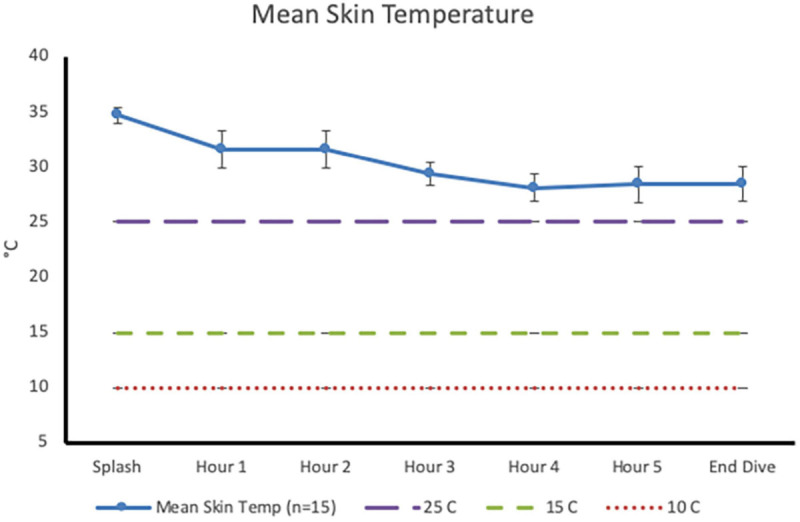
Mean skin temperature (mean ± SD) at dive splash and each subsequent hour of the dive. Dashed lines identify temperature of 25°C (minor performance impairments), 15°C (sharp decrease in finger dexterity) and 10°C (risk for non-freezing cold injuries).

**FIGURE 3:**
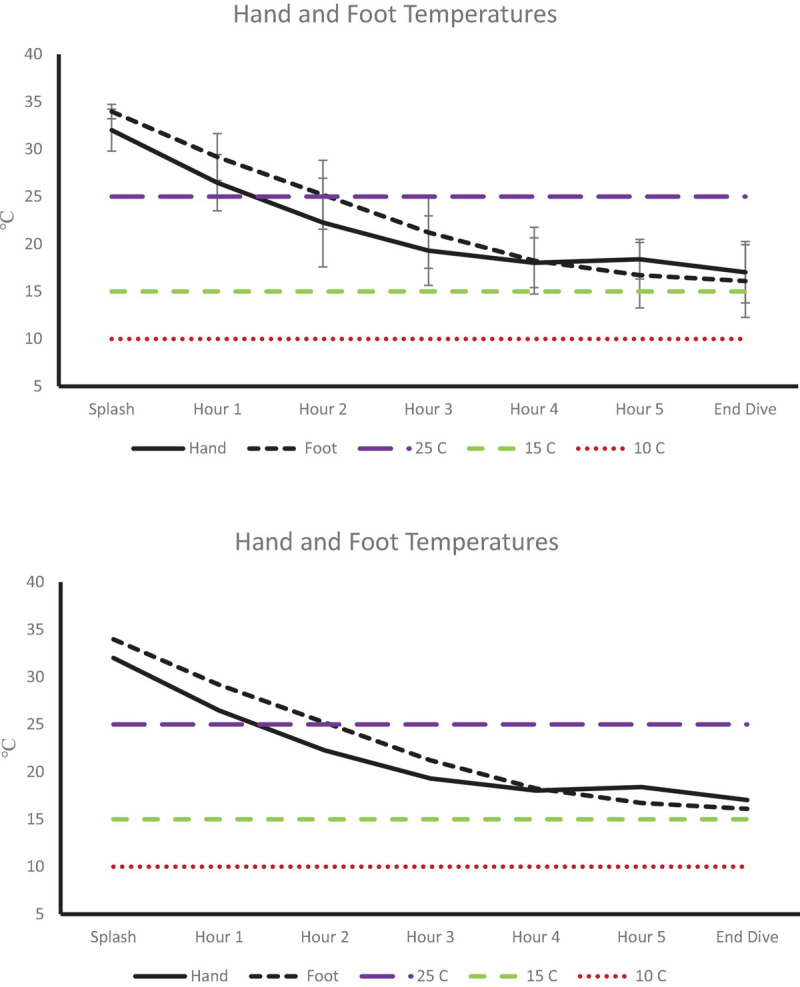
Extremity temperatures at dive splash and each subsequent hour of the dive. Dashed lines identify temperature of 25°C (minor performance impairments), 15°C (sharp decrease in finger dexterity) and 10°C (risk for non-freezing cold injuries).

Post-dive, core temperature (36.8 ± 0.4°C) was significantly lower than pre-dive (37.5 ± 0.4°C; *p* < 0.001, 95% CI [0.4, 0.9], Cohen’s *d* = 1.6; Figure 1). Post-dive, mean skin temperature (28.5 ± 1.6°C) was significantly lower than pre-dive (34.7 ± 0.7°C; *p <* 0.001, 95% CI [5.4, 7.0], Cohen’s *d* = 4.1; Figure 2). Hand and foot temperatures dropped 15 and 17°C colder, respectively, at the end of the dive compared to dive splash. Hand temperature decreased significantly from 32.0 ± 2.2°C at dive splash, to 17.0 ± 3.0°C at the end of the dive, *p* < 0.001, 95% CI [12.7, 17.1], Cohen’s *d* = 3.8. Foot temperature decreased significantly from 34.0 ± 0.8°C at dive splash to 16.1 ± 3.8°C at the end of the dive, *p* < 0.001, 95% CI [15.9, 19.9], Cohen’s *d* = 4.8. All extremity temperatures were significantly colder at each hour of the dive compared to dive splash, *p* < 0.001 (Figure 3).

### Metabolic Rate

**FIGURE 4:**
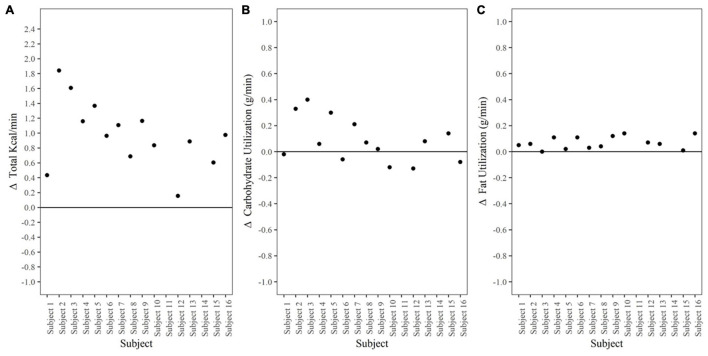
Metabolic Rate and Substrate Utilization. **(A)** Δ Metabolic rate (kcal/min) pre-dive to post-dive. Data points represent individual divers. **(B)** Δ Carbohydrate utilization (g/min) pre-dive to post-dive. Data points represent individual divers. **(C)** Δ Fat utilization (g/min) pre-dive to post-dive. Data points represent individual divers.

Data shown in Figure 4A represent Δ metabolic rate (kcal/min) pre-dive to post-dive for individual divers. On average, metabolic rate (kcal/min) during the post-dive measurement period was different than prior to the dive, increasing from 1.9 ± 0.2 kcal/min pre-dive to 2.8 ± 0.2 kcal/min post-dive, *p* < 0.001, 95% CI [0.7, 1.2], Cohen’s *d* = 2.2 (Table 2). Change in metabolic rate in response to the prolonged cold-water dive, and post-dive metabolic rate were not correlated to body fat percentage, lean body mass, BSA, or body temperature, *p* > 0.05.

**TABLE 2:** Metabolic and physiologic responses.

	**n**	**Pre dive mean ± S**	**Post dive mean ± SD**	**Pre to post Δ**	**95% CI Δ**	**Cohen’s *d***
VO_2_ (L/min)	15	0.40 ± 0.04	0.60 ± 0.10*	0.21	0.2, 0.3	2.5
VO_2_ (mL/kg/min)	15	4.3 ± 0.5	6.7 ± 1.0*	2.4	1.8, 2.9	2.5
Kcal/min	14	1.9 ± 0.2	2.8 ± 0.2*	1.0	0.7, 1.2	2.17
RER	14	0.90 ± 0.06	0.87 ± 0.04	0.03	−0.01, 0.10	0.5
Fat use (g/min)	14	0.07 ± 0.03	0.13 ± 0.04*	0.06	0.01, 0.09	0.8
CHO use (g/min)	14	0.30 ± 0.10	0.40 ± 0.20	0.08	0.01, 0.25	0.6
Tcore (°C)	15	37.5 ± 0.4	36.8 ± 0.4*	0.7	0.4, 0.9	1.6
T_*msk*_ (°C)	15	34.7 ± 0.7	28.5 ± 1.6*	6.2	5.4, 7.0	4.1
Cold strain index	15	N/A	2.6 ± 1.2	N/A	N/A	

*Different than pre-dive, p < 0.05.

*Data presented are mean ± standard deviation.*

*VO_2_, oxygen uptake; kcal, kilocalorie; min, minute; RER, respiratory exchange ratio; g, gram; CHO, carbohydrate; Tcore, core temperature; Tmsk, mean skin temperature.*

*Where n < 16, data was compromised and unable to be analyzed.*

### Substrate Utilization

Substrate utilization during the post-dive measurement period was 57.5% carbohydrate, 42.5% fat; RER 0.87 ± 0.04. This did not differ from the pre-dive measurement period, *p* = 0.10, 95% CI [−0.01, 0.02], Cohen’s *d* = 0.5 (Table 2).

Carbohydrate utilization (g/min) Δ pre-dive (0.30 ± 0.10) to post-dive (0.40 ± 0.20) was not different, *p* = 0.08, 95% CI [−0.2, 0.01] (Figure 4B). Fat utilization (g/min) Δ pre-dive to post-dive increased significantly from 0.07 ± 0.04 g/min pre-dive, to 0.13 ± 0.04 g/min post dive, *p* < 0.001, 95% CI [0.04, 0.10], Cohen’s *d* = 1.4 (Figure 4C). Substrate utilization was not correlated to body fat percentage, lean body mass, BSA, or body temperature; *p* > 0.05.

### Heart Rate

Heart rate (Table 3) data was separated into two phases, donning gear and during the dive. Heart rate during jock up (111 ± 12) was significantly higher than during the dive (81 ± 8), *p* < 0.001. While submerged, heart rate maintained between 50 and 59% of divers’ age predicted max (220-age).

**TABLE 3:** Heart rate and energy expenditure*.

	**n**	**Heart rate (bpm) average ± SD**	**Heart rate (bpm) range**	**Calories (kcal) average ± SD**	**Calories (kcal) range**
Donning gear	6	111 ± 12	91–129	279 ± 92	153–394
Dive	13	81 ± 8	65–92	1,223 ± 348**	699–1,586**

*Provided from Polar Team Pro app.

**n = 7.

## Discussion

Thick neoprene wetsuits provided effective thermoprotection for divers for 6-h in 5°C water. Adequate thermal insulation maintained core temperature in combination with a 53% increase in metabolic rate (kcal/min), regardless of the divers’ body composition. These data provide valuable information on the thermoregulatory and metabolic demands of NSW divers during a prolonged training dive in cold-water that required full submersion. The data presented herein are relevant not only for military operations but for commercial and recreational divers that face extreme environmental conditions as part of their occupation or sport.

A critical aspect of cold-water diving is prevention of hypothermia through proper gear as well as generation of metabolic heat. There is the assumption that wetsuits provide adequate protection by design (Naebe et al., 2013); however, most recreational dives last 45–60 min which is not representative of military dive times. There are only a few studies that have addressed prolonged cold-water diving in military personnel and no data is available in recreational divers (Vaughan and Strauss, 1975; Riera et al., 2014). Our data align with these previous efforts in that core temperature slightly decreased at the onset of the dive, yet was preserved, overall decreasing −0.7°C in our data compared to −0.9 to 1.2°C in the earlier studies. Circadian fluctuations have been reported to effect absolute core temperature by ∼0.5°C over a 24-h period (Refinnetti and Menaker, 1992); however, this variation is only critical during exercise at a fixed rate of heat production (Ravanelli and Jay, 2021). As this effort was passive, it is unlikely that core temperature variation was a function of circadian cycle. Thus, the variation may be attributed to differences in body composition, body size, and wetsuit thickness between the divers in our study and those previously reported.

The cold environment also necessitated an increase in metabolic rate (kcal/min) to maintain thermal balance. Divers caloric expenditure approached similar reports of metabolic rate reaching 3–5 kcal/min during cold-induced shivering (Castellani and Young, 2016), and metabolic heat increases of 58–83% in head out water immersion (Marino et al., 1998) despite the fact that the divers in our study were wearing highly insulative wetsuits and were completely submerged. Metabolic rate increased in every diver, reaching ∼3 kcal/min (∼2 METs) post-dive with minimal physical exertion; heart rate ranged between 50 and 59% of age predicted maximum for majority of the dive. The increase in metabolic rate compared to pre-dive was driven by an overall increase in fat oxidation as there was no statistical difference in carbohydrate utilization pre-dive to post-dive. However, carbohydrate provided 57.5% of substrate for energy production during the post-dive period. Theoretically, assuming oxidation rates during the post-dive measurement period accounted for the 6-h dive, total fat oxidation was 47 g (423 kcal) compared to 144 g carbohydrate (576 kcal). This is consistent with other cold exposure literature in which carbohydrate is the preferred fuel especially when shiver rates are high (Horvath et al., 1956; Martineau and Jacobs, 1988; Vallerand and Jacobs, 1989; Marino et al., 1998; Haman and Blondin, 2017) and individuals are not habituated to the cold (Castellani and Young, 2016).

While our data show a universal increase in metabolic rate with a wide variation in both absolute and relative rates; these metabolic adjustments were not correlated to body composition or body temperature as noted in immersion efforts. In the present effort, the two leanest divers (14% body fat) displayed one of the lowest (Δ0.43 kcal/min from pre-dive) and the highest (Δ 1.61 kcal/min from pre-dive) metabolic rate change. Moreover, body composition was not correlated to change in core temperature. Individuals with larger body surface area and lower body fat level are expected to have a large drop in core temperature necessitating a higher increase in metabolic rate. Previous immersion work demonstrated that there are distinct differences in metabolic rate dependent upon body composition with lean men (∼12%) showing greater increase in caloric expenditure over time as compared to men with body fat between 18 and 22% (Glickman-Weiss et al., 1995; Wakabayashi et al., 2006). Similarly, in lean military divers comparable to those in this effort, there was a greater increase in metabolic rate despite water temperatures being much greater than in the current study (Riera et al., 2014). These variations may be partially explained by slight differences in thermoprotective gear thickness and technology as well as individual differences in thermoregulation. Diversity in the climates of geographic regions where participants grew up along with their military history culminates a mix of prior exposure to the cold. Past experiences, including both chronic prolonged cold exposure, and chronic exposure to repeated, intermittent cold can provoke varied physiologic adjustments in response to cold (Davis, 1961; Golden and Tipton, 1988; Young, 1996; Daanen and Van Marken Lichtenbelt, 2016). Castellani and Young (2016) identifies the different phases of progressive development of physiological response to cold to be a function of cold habituation, metabolic adaptation, and insulative properties. We suspect physiologic adjustments from prior cold exposure resulted in the variety of metabolic responses recorded given variation in insulative properties (body composition) did not account for metabolic differences. Nonetheless, it highlights the need for more work in the context of thermoregulation and subsequent effect on metabolism while in thermoprotective gear.

The greatest risk in cold-exposure is non-freezing cold injuries to the extremities resulting in permanent nerve damage (Havenith et al., 1995; Imray et al., 2009). Peripheral vasoconstriction occurs to shunt blood to the core at the expense of the periphery, especially the hands and feet. Mean skin temperature gradually decreased until hour three, where it plateaued until cessation of the dive at hour six. This patterns paralleled the findings in of Riera et al. (2014) which is the only analogous effort we could find. As anticipated, hand and foot temperatures declined throughout the dive. Most notably, by hour two, hand temperature dropped to below 25°C, which increases risk to manual dexterity (Cheung et al., 2003), albeit above threshold for non-freezing cold injuries (10°C; Imray et al., 2009; Castellani, 2011). This is in contrast to immersion data, where peripheral temperature rapidly and continuously decreased (Hayward and Keating, 1979). Data from cold-water immersion and cold-exposure studies suggest that the regulation of thermogenesis is a function of cold exposure; with the majority of these efforts having been conducted in individuals immersed between 60 and 180 min and in a nude or semi-nude state in a laboratory environment with water temperatures between 10 and 20°C (Martineau and Jacobs, 1988, 1989; Glickman-Weiss et al., 1995; Castellani et al., 1998; Sramek et al., 2000). The primary focus of early efforts was to understand thermoregulation in the event of accidental exposure, i.e., falling off a vessel, falling through a frozen lake to determine survivability in extreme situations (Hamlet, 1976; Hayward and Eckerson, 1984). From both case reports and controlled lab studies, predictive models on thermoregulatory response have contributed guidance to operational safety (Hayward and Eckerson, 1984; Tipton et al., 1991; Harman and Herndon, 2006; Xu et al., 2007). While these efforts have been critical in our understanding of time to hypothermia and risk of non-freezing cold injuries they lack the application to the military dive community that often operates in colder water temperatures and for longer durations of time. Moreover, these efforts do not address the caloric needs of these individuals in order to sustain training and operations. While the assumption that thermoprotective gear preserves core temperature and protects the periphery is valid as verified in the current effort; inter-individual differences in metabolic adjustments has not been well explored. Moreover, to our knowledge the current effort is the first to document thermoregulation in military divers in extremely cold water temperature and at extended dive time in wet-suits versus the classic dry-suit typically worn.

There are several limitations that need to be addressed with respect to the current effort. Primarily, the data were collected during active training and thus could not interfere with the training goals. Moreover due to the specialized nature of training it was not possible to have a comparative control group. Indirect calorimetry was performed pre-dive and post-dive, as divers were required to used their standard issued dive rig during training for safety issues. As such, there may be different findings if open circuit dive apparatus are used with respect to thermoregulation and/or metabolic burden Pre-dive gas collection was performed in a morning fasted state; however, respiratory exchange ratio calculated from the expired gas collection is reflective of oxidative metabolism of carbohydrate and fatty acids and does not account for glucose used in non-oxidative glycolysis, or amino acid metabolism. Diet was not controlled for as it was intended for the effort to be reflective of “real world” scenario and we wanted the divers to fuel themselves as they normally would pre-dive as to not induce a variable that may impact performance. Metabolic metrics are therefore representative of individual’s dietary habits. Dietary recall was not performed due to time and wide variation in dietary pattern. Divers were allowed to eat following resting metabolic measurement; however, the majority chose not to. This may have impacted post macronutrient measurements slightly (Flatt et al., 1985; Abtahi et al., 2015; Gribok et al., 2016). Moreover, women were not included in this effort as they were not assigned to the training and thus the results from this effort can only be applied to male population. Finally, this effort was conducted in elite military divers and thus results may only be relevant to a fit population. Further work is needed to determine whether there are similar results in an overweight and/or obese population.

In summary, this effort highlights the fact that metabolic demand increases 53% during prolonged, extreme cold submersion despite thermal protection. The rise in metabolic rate was attributed to an overall increase in absolute fat oxidation, with carbohydrate use being the primary relative fuel source over time. This change was independent of anthropometric characteristics and fitness levels of divers. Thermoprotective gear and increased metabolism preserved core temperature and likely slowed the cooling of the periphery. Inter-individual variability in metabolic and substrate use emphasize the need for more work in this area in order to create generalizable data that can be used not only in military populations but for rescue and commercial divers that operate and work in austere environments. Variation in thermoregulation, not explained by body composition, highlight the need to understand habituation and metabolic adaptation prior cold exposure; an area of research that is currently not well understood. Continued characterization of metabolic demands during these training scenarios will help guide individual nutritional interventions, optimize mission planning, and enhance operational readiness.

## Data Availability Statement

The raw data supporting the conclusions of this article will be made available by the authors, without undue reservation.

## Ethics Statement

The studies involving human participants were reviewed and approved by the Naval Health Research Center-Institutional Review Board protocol number NHRC.2017.0019. The patients/participants provided their written informed consent to participate in this study.

## Author Contributions

KK received the funding, designed the study, finalized the manuscript prior to submission, and was in charge of overall project management, analysis, and finalization of manuscript. LA collected the data. AC analyzed the data in consultation with JB. AC wrote the manuscript with support from LA and KK. All authors contributed to the article and approved the submitted version.

## Author Disclaimer

KK a military service member or employee of the U.S. Government. This work was prepared as part of my official duties. Title 17, U.S.C. §105 provides that copyright protection under this title is not available for any work of the U.S. Government. Title 17, U.S.C. §101 defines a U.S. Government work as work prepared by a military service member or employee of the U.S. Government as part of that person’s official duties. Report was supported by Office of Navy Research under work unit no. N1506. The views expressed in this article are those of the authors and do not necessarily reflect the official policy or position of the Department of the Navy, Department of Defense, nor the U.S. Government. The study protocol was approved by the Naval Health Research Center-Institutional Review Board in compliance with all applicable Federal regulations governing the protection of human subjects. Research data were derived from an approved Naval Health Research Center-Institutional Review Board protocol number NHRC.2017.0019.

## Conflict of Interest

AC, LA, and JB were employed by company Leidos, Inc. The remaining author declares that the research was conducted in the absence of any commercial or financial relationships that could be construed as a potential conflict of interest.

## Publisher’s Note

All claims expressed in this article are solely those of the authors and do not necessarily represent those of their affiliated organizations, or those of the publisher, the editors and the reviewers. Any product that may be evaluated in this article, or claim that may be made by its manufacturer, is not guaranteed or endorsed by the publisher.
